# Gonadotropin-Releasing Hormone Agonist Corrects Defective Mini-Puberty in Boys with Cryptorchidism: A Prospective Randomized Study

**DOI:** 10.1155/2018/4651218

**Published:** 2018-07-03

**Authors:** Beata Vincel, Gilvydas Verkauskas, Vytautas Bilius, Darius Dasevicius, Dalius Malcius, Birute Jones, Faruk Hadziselimovic

**Affiliations:** ^1^Children's Surgery Centre, Clinic of Gastroenterology, Nephrourology and Surgery, Institute of Clinical Medicine, Faculty of Medicine, Vilnius University, Vilnius, Lithuania; ^2^Children's Surgery Centre, Faculty of Medicine, Vilnius University, Vilnius, Lithuania; ^3^National Centre of Pathology, Affiliate of Vilnius University Hospital Santaros Klinikos, Vilnius, Lithuania; ^4^Department of Pediatric Surgery, Lithuanian University of Health Sciences, Kaunas, Lithuania; ^5^Children's Hospital, Affiliate of Vilnius University Hospital Santaros Klinikos, Vilnius, Lithuania; ^6^Cryptorchidism Research Institute, Kindermedizinisches Zentrum Liestal, Liestal, Switzerland

## Abstract

**Introduction:**

This prospective study investigated the efficacy of a gonadotropin-releasing hormone agonist (LH-RHa) in restoring defective mini-puberty.

**Materials and Methods:**

Boys with isolated bilateral cryptorchidism and defective mini-puberty were randomly divided into two groups. The “surgery only” group underwent a second orchidopexy without hormonal treatment (control). The “LH-RHa” group received LH-RHa therapy followed by a second orchidopexy. The number of Ad spermatogonia and the total germ cell count per tubule (S/T) were analyzed.

**Results:**

Five boys were included in each arm. In the LH-RHa group, the median S/T increased from 0.11 to 0.42, p=0.04. In the surgery only group, the median S/T did not change. In the surgery only group, none of the testes had Ad spermatogonia. In contrast, in the LH-RHa group, all testes completed the transition from gonocytes to Ad spermatogonia (p=0.008).

**Conclusions:**

Treatment with LH-RHa was effective in rescuing defective mini-puberty in boys with bilateral cryptorchidism.

## 1. Introduction

Cryptorchidism is one of the most common urogenital endocrinopathy conditions in newborn boys, with an incidence of 1.8-4.1% [1]. It is associated with infertility, and it is one of the main etiologic causes of nonobstructive azoospermia in men [2]. To preserve future fertility, the current consensus recommends treating cryptorchidism with orchidopexy during the first year of life [3]. However, successful scrotal repositioning of the testis does not prevent infertility, even when the surgery is performed early in life [4–6]. The low fertility potential in men with surgically treated cryptorchidism is asserted to be hormonal in origin; it arises as a consequence of impaired “mini-puberty”, a period when gonocytes translate into adult (dark) spermatogonia [7]. In male infants, mini-puberty occurs between two and four months after birth, when an increase in gonadotropin secretion stimulates Leydig cells to secrete testosterone [8, 9]. This testosterone increase is blunted in infants with cryptorchidism [10–13], and it results in an inadequate transition of gonocytes into adult dark (Ad) spermatogonia [7, 14]. The importance of these developmental changes in the testes during mini-puberty was underscored in a 20-year, long-term follow-up prospective study, which showed that, following successful surgery, establishing fertility is largely dependent on normal mini-puberty and, as a result, the presence of Ad spermatogonia [15]. Therefore, the achievement of normal fertility following successful surgery depends mainly upon the presence of Ad spermatogonia at the time of orchidopexy [15, 16].

The present prospective, randomized study aimed to prove the hypothesis that gonadotropin-releasing hormone agonist (LH-RHa) could rescue defective mini-puberty and induce gonocytes to transition into Ad spermatogonia.

## 2. Patients and Methods

Boys up to 6 years old with isolated bilateral cryptorchidism that underwent orchidopexy in a Pediatric Surgery Center were prospectively included in the study. Boys were excluded when they exhibited any syndromes, associated anomalies, or known hypogonadotropic hypogonadism, any previous hormonal treatment, or retractile testes. During the first orchidopexy, testicular biopsies of ipsilateral testicle were obtained from all patients. Boys with high infertility risk (no Ad spermatogonia in the testicular biopsy) were randomly divided into two groups. The “surgery only” group underwent a second orchidopexy without hormonal treatment. The “LH-RHa treatment” group received an intranasal Buserelin spray (10 *μ*g), delivered every second day in the evening for 6 months, followed by a second orchidopexy. This dose of Buserelin was selected based on the pharmacokinetic study performed by Sandow et al. [17]. Another biopsy was acquired from both groups during the second surgery. Based on the findings from the second biopsy, no Ad spermatogonia, participants from the “surgery only” group received identical adjuvant hormonal treatment as “LH-RHa treatment” group. All these patients will be followed until adolescence. The positions of the testicles (abdominal cavity or inguinal channel) were evaluated during surgery.

### 2.1. Randomization

We used a computer program for randomization. It displayed two numbers (1 and 2) in random succession, which were used to allocate the sample of 20. Every new patient that consented to participate in the study was allocated in alternating succession to the surgery only or the treatment group.

### 2.2. Testicular Biopsy

**Figure 1:**
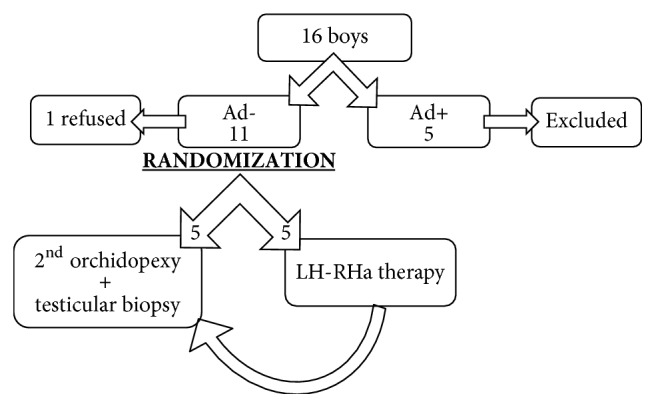
Flow chart shows the selection of study participants.

Each testis located in the cryptorchid position was biopsied once at the time of orchidopexy. Biopsies were performed using surgical loops. Rice grain size testicular tissue was obtained at the most lateral testicular curvature opposite epidydimis—an area known to have the less blood supply. Specimens were fixed in 3% glutaraldehyde and embedded in Epon. Semi-thin sections, 1-*μ*m thick, were stained with toluidine blue and examined with light microscopy at a magnification of 400×. We analyzed the number of Ad spermatogonia and the number of germ cells per at least 100 tubular cross-sections (S/T). The biopsy materials from all patients were histologically examined by a pathologist (DD) experienced in analyzing semi-thin sections of prepubertal testes. The examinations were double checked by the senior author (FH). In prepubertal testis, Ad spermatogonia are identified according to criteria first published in 1974 by Seguchi and Hadziselimovic [18]. This type of germ cell has a typical halo in the nucleus, called the rarefication zone, and the cytoplasm has a darker appearance [18]. Patients with Ad spermatogonia in the first biopsy were excluded from the present study (Figure 1).

### 2.3. Statistical Analysis

Statistical analysis of the results was performed with the open access, statistical program “R”. Nonparametric methods were used. The Wilcoxon signed-rank test was used to compare medians. Fisher's exact test was used to compare categorical values. Differences between groups were considered statistically significant, when p<0.05.

### 2.4. Ethics

This study was conducted in accordance with the Helsinki Declaration, and it was approved by the Vilnius Regional Biomedical Research Ethics Committee, No. 158200-580-PPI-17. Written informed consent was obtained from both parents of each child.

## 3. Results

Parents of 16 boys with bilateral cryptorchidism agreed to allow the boys to participate in the study. Five boys had Ad spermatogonia in the first biopsy and were excluded (Figure 1). Parents of one boy lacking Ad spermatogonia in the first biopsy refused to continue the study for personal reasons. Five boys in each arm completed the study, and both biopsies were analyzed. At the time of the first surgery, randomized patients were aged 7 to 55 months. The two groups were age-equivalent (medians: 20 and 22 months; p=0.3173). At the time of the second surgery, patients were aged 10 to 62 months, and there was no significant age difference between groups (medians: 27 and 30 months; p=0.1797).  Table 1 summarizes the biopsy data and the testicular positions at surgery.

Treatment was generally well tolerated. One patient disliked nasal administration, due to an apparent burning sensation, which led to the discontinuation of administration after 1 month of treatment. Nevertheless, the 1-month treatment provided positive effects on fertility potential: the S/T was 0.08 in the first biopsy and 0.13 in the second biopsy, and Ad spermatogonia appeared. No other side effects were recorded. Thus, all other patients completed the six-month treatment.

**Figure 2:**
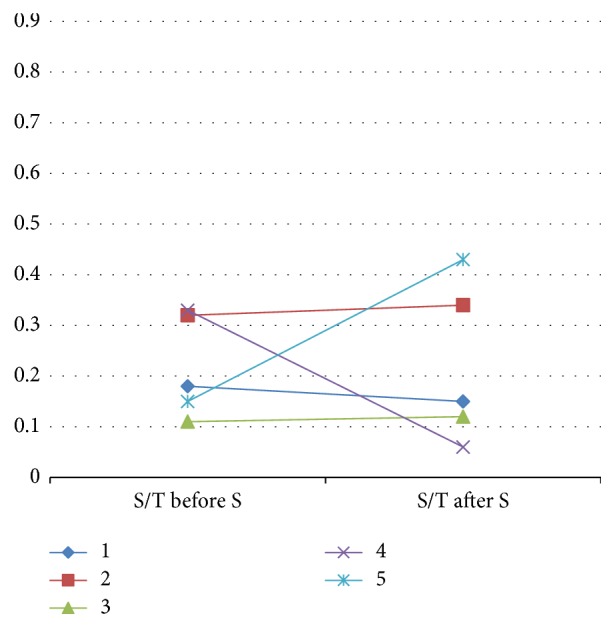
S/T changes in the “surgery only” group. Each symbol and color represents one patient. S: surgery.

**Figure 3:**
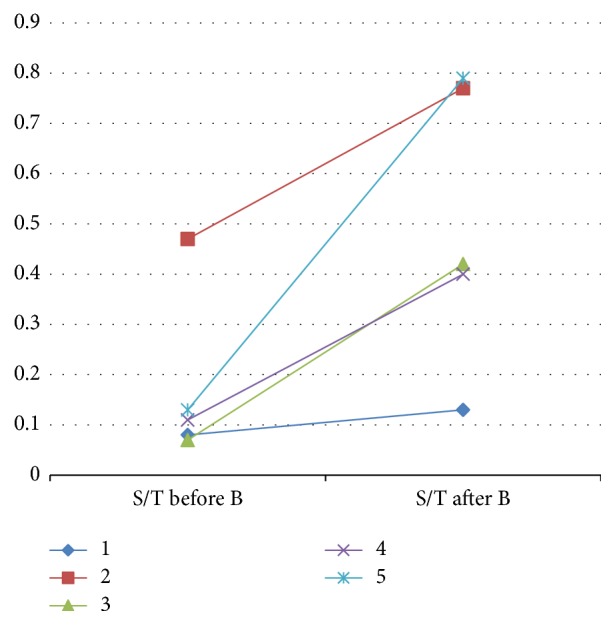
S/T changes in the “treatment” group. Each symbol and color represents one patient. B: Buserelin.

At the first surgery, the S/T was similar between the two groups (p=0.67). S/T changes in both groups are shown in Figures 2 and 3. In the surgery only group, the S/T only slightly increased in two patients, it decreased in two others, and one patient exhibited a clear S/T increase. In contrast, in the treatment group, the median S/T significantly increased from 0.11 to 0.42 (p=0.03, paired-samples Wilcoxon test, one-tailed). Ad spermatogonia appeared only in patients who received hormonal treatment (p=0.008; Fisher test, 2-tailed).

## 4. Discussion

To the best of our knowledge, the present study was the first prospective randomized study to analyze histological findings of boys with bilateral cryptorchidism after a 6-month LH-RHa treatment. We found that LH-RHa increased the number of germ cells and, more importantly, induced the complete transition of gonocytes and fetal spermatogonia into Ad spermatogonia. These results indicated that this treatment sufficiently stimulated the pituitary gonadal axis to induce the development of Ad spermatogonia—a stem cell for future sperm production (Table 1). Our results were consistent with previous observations from open studies and supported the hypothesis that GnRHa could increase testosterone levels and rescue the physiological function of “mini-puberty” [7, 14]. In a previous study, 86% of patients at increased risk of infertility (i.e., patients that lacked Ad spermatogonia in their testes) exhibited a normal sperm count after LH-RHa treatment [16]. Thus, the effect of LH-RHa treatment persisted into adulthood. It was also shown previously that induction of germ cell proliferation could be achieved with native LH-RH [19, 20] and with nafarelin [21]. In a small open study, nafarelin treatment improved the total germ cell count in 75% of cryptorchid testes and in 83% of contralateral descended gonads [21]. Biers and Malone analyzed the level of evidence for improved fertility indices, semen analyses, and paternity rates, following hormonal therapy in males with undescended testes [22]. They concluded that adjuvant GnRH therapy was safe and efficacious for boys with cryptorchidism, particularly bilateral cases [22]. The results of our randomized study supported these key findings.

Although the technique of testicular biopsy has been refined for decades, the possible damage to testicle is still frequently debated. It has been shown conclusively that performing biopsy in prepubertal testes does not cause any damage to the developing gonad and may be beneficial in diagnosing high infertility risk, as well as Ca in situ [23, 24].

A critical issue in any analysis, particularly when working with human samples, is the number of cases that are included. In this study, we included 10 patients enrolled sequentially and they received treatment based on a randomized allocation. Thus, the decision to give or not to give hormone treatment was unbiased by any parameter. Five testicular biopsies from the LH-RHa treatment group were compared to five samples from the surgery only group. Although the sample size was small, it was sufficient for discerning statistical differences. Long term studies comparing semen samples of randomly allocated boys and larger studies with different regiments of treatment appear to be advocated.

The European Association of Urology and the European Society for Paediatric Urology published guidelines for managing undescended testes. They recommended hormonal treatment with LH-RHa to preserve the fertility potential of boys with bilateral cryptorchidism [25]. Our data supported this suggestion. The evidence was sufficiently strong to recommend a change in current clinical practice for treating cryptorchidism.

A previous study was conducted at the molecular level to profile RNA extracted from biopsy samples acquired from the first four patients from this randomized study before and after LH-RHa treatment. From detected 28,645 transcripts, 6,469 showed a significant difference in expression levels; expression increased in 5,823 cases (90%) following LH-RHa treatment (False Detection Rate <0.05; absolute expression change ≥ 2-fold). RNA profiling revealed that these GnRHa-induced transcriptional response involved genes that controlled the hypothalamus-pituitary-gonadal axis and reproduction and testosterone synthesis via both the classical and alternate pathways. The development of Ad spermatogonia was rescued with LH-RHa treatment. Furthermore, that analysis showed that several lncRNAs involved in epigenetic programming were responsive to GnRHa treatment. In contrast, surgery alone had no effect on gene expression. Those studies pointed to the molecular mechanisms that underlie the ability of GnRHa to rescue fertility and underscored the importance of GnRHa treatment in rescuing fertility [26].

## 5. Conclusion

The present study showed that LH-RHa therapy corrected impaired mini-puberty in boys with bilateral cryptorchidism. Further studies are advocated.

## Figures and Tables

**Table 1:** Summary of the study data.

Age, months	Position of the testicle		Ad/T1	Ad/T2	S/T1	S/T2
	1st operation	2nd operation				
SURGERY ONLY GROUP						

10	Peritoneal cavity	Peritoneal cavity	0	0	0.18	0.15

7	Peritoneal cavity	Inguinal channel	0	0	0.32	0.34

20	Inguinal channel	Inguinal channel	0	0	0.11	0.12

22	Peritoneal cavity	Peritoneal cavity	0	0	0.33	0.06

53	Inguinal channel, upper 1/3	Inguinal channel	0	0	0.15	0.43

LH-RHa TREATMENT GROUP						

13	Inguinal channel, at external ring	Ectopic, perineal position	0	00085	0.08	0.13

32	Inguinal channel	Inguinal channel	0	0.0109	0.47	0.77

55	Inguinal channel, upper 1/3	Inguinal channel, upper 1/3	0	0.0056	0.07	0.42

22	Inguinal channel, upper 1/3	Inguinal channel	0	0.005	0.11	0.40

10	Inguinal channel, at external ring	Inguinal channel	0	0.0208	0.13	0.79

Ad/T1: adult dark spermatogonia per tubular cross-section in the first biopsy.

Ad/T2: adult dark spermatogonia per tubular cross-section in the second biopsy.

S/T1: germ cells per tubular cross-section in the first biopsy.

S/T2: germ cells per tubular cross-section in the second biopsy.

## Data Availability

Study data is available upon request sent to the corresponding author.
